# Blueberry Polyphenol Extracts Enhance the Intestinal Antioxidant Capacity in Weaned Rats by Modulating the Nrf2–Keap1 Signal Pathway

**DOI:** 10.3389/fphys.2021.640737

**Published:** 2021-02-04

**Authors:** Fangfang Zhao, Shen Yan, Mengliang Tian

**Affiliations:** ^1^Chengdu Diversity Co. Ltd., Chengdu, China; ^2^Agricultural Information Institute, Chinese Academy of Agricultural Sciences, Beijing, China; ^3^College of Agronomy, Sichuan Agricultural University, Chengdu, China

**Keywords:** blueberry, antioxidant status, inflammation, small intestine, weaned rats

## Abstract

Weaning causes the generation of excessive reactive oxygen species in the body, which could lead to oxidative stress. Polyphenols, for which blueberries are an important dietary source, are known for various health benefits including antioxidant properties. Here, we sought to elucidate the effects of blueberry polyphenol extracts (BPE) on intestinal antioxidant capacity and possible underlying mechanisms in weaned rats. Ninety-six rats were assigned to two groups and fed either a standard diet or a standard diet supplemented with BPE (200 mg/kg). The results showed that BPE supplementation increased (*P* < 0.05) catalase and superoxide dismutase activities and decreased (*P* < 0.05) interleukin-1 and interferon-γ contents in the jejunum and ileum. The abundances of mammalian target of rapamycin, ribosomal p70 S6 kinase and eukaryotic initiation factor 4E-binding protein 1 mRNA were elevated in the jejunum and ileum (*P* < 0.05) after BPE supplementation. Additionally, BPE supplementation decreased (*P* < 0.05) Kelch-like ECH-associated protein 1 (Keap1) gene transcription and enhanced (*P* < 0.05) NF-E2-related factor 2 (Nrf2) gene transcription in the jejunum and ileum. According to our results, BPE-induced protective effects against oxidative stress appear through the promotion of the jejunal and ileal antioxidant defense system in weaned rats, which was associated with the Nrf2–Keap1 signaling pathway.

## Introduction

Weaning can have many negative effects, including enhanced disease susceptibility (Yin et al., 2013), impaired intestinal functions (Xiong et al., 2015), as well as reduced feed intake and suppressed growth (Campbell et al., 2013). Moreover, weaning also generates excessive reactive oxygen species in the body (Yin et al., 2014), which leads to further oxidative stress (Han et al., 2011). A recent study found that suitable therapies and nutritional support (e.g., polyphenols) can provide beneficial effects to relieve the problems associated with weaning (Ruan et al., 2014).

Blueberry is widely consumed as a health food; its major bioactive components are phenolic acids, ascorbic acid, flavonol, anthocyanins, and tannins (Szajdek and Borowska, 2008; Nile and Park, 2014). Recent studies have shown that the bioactive components of blueberry possess anti-oxidative, anti-inflammatory and anti-cancer effects (Kausar et al., 2012; Skrovankova et al., 2015; Cásedas et al., 2017). As such, the functional components of blueberry are anticipated to be beneficial in both preventing and treating oxidative stress-related diseases (Huang et al., 2016; Wu et al., 2017). However, no study has yet indicated the effects of blueberry polyphenol extracts (BPE) on the antioxidant status of the small intestine in weaned rats. Hence, the changes in antioxidant status in the small intestine of weaned rats after BPE supplementation need to be elucidated.

At present, we evaluate the effects of dietary BPE supplementation on the antioxidant status in the small intestine of weaned rats, as well as the underlying mechanisms. Our results can illuminate the relationship between BPE and human health and pave the way for the development of BPE as a functional antioxidant.

## Materials and Methods

### Preparation of BPE

Homogenized blueberry pulp was extracted with 200 mL ethanol solution for 120 min at 50°C with ultrasonic-assisted extraction. Subsequently, the extraction mixture was filtered by vacuum filtration to obtain the crude blueberry extracts. Next, the crude blueberry extracts were purified by XAD-7 macroporous resin, and then freeze-dried into powder (Jiao et al., 2019).

### Animal Experiments

Ninety-six Sprague-Dawley rats (weaned at 21 days) weighing 60–65 g were allocated to two groups with eight pens per group (six rats per pen). The groups were as follows: (1) standard diet (CON group) and (2) standard diet + 200 mg/kg BPE (BPE group). Diets were formulated on the basis of the AIN-93G requirements for the growth phase of laboratory rodents (Reeves et al., 1993). Throughout the 2-week experiment, the temperature ranged from 24 to 28°C and a 12 h light/dark cycle was maintained. All rats were given free access to feed and water.

### Growth Performance

At the start and end of the trial, each rat's body weight (BW) was assessed, and the feed intake was recorded daily, to calculate the average daily gain (ADG) and average daily feed intake (ADFI).

### Sample Collection

After feeding for 14 days, one rat was chosen from each pen based on the average BW, anesthetized with an intravenous injection of sodium pentobarbital (200 μg/g BW), and then euthanised by exsanguination. Immediately after slaughtering, jejunum and ileum samples were obtained from each rat and then stored at −80°C until analysis.

### Tissue Preparation

Frozen jejunum and ileum samples (0.1 g) were rapidly weighed, thawed and homogenized in a nine-fold volume (w/v) of physiological saline and then centrifuged at 3,000 × g, 4°C, for 10 min. Thereafter, the supernatant was collected and then stored at −20°C for further assay.

### Antioxidant Capacity Assay

Malondialdehyde (MDA), superoxide dismutase (SOD), catalase (CAT), and total antioxidant capacity (T-AOC) in the jejunum and ileum were assayed using corresponding detection kits (Nanjing Jiancheng Bioengineering Institute, Nanjing, China). All antioxidant-related indices were spectrophotometrically tested using a microplate reader (SpectraMax M2, Molecular Devices, USA) in accordance with the manufacturer's instructions.

### Enzyme-Linked Immunosorbent Assay

The contents of interleukin-1 (IL-1), IL-6, tumor necrosis factor-α (TNF-α), and interferon-γ (IFN-γ) were measured using commercial kits according to the manufacturer's instructions (Jiangsu Jingmei Biotechnology Co., Ltd., Yancheng, China).

### Total RNA Isolation and Reverse Transcription

Approximately 0.1 g of frozen jejunal and ileal samples were homogenized in 1 mL of RNAiso Plus (Takara, Dalian, China) according to the manufacturer's instructions to extract total RNA. The yield and quality of the total RNA were estimated by a nucleic-acid/protein analyser (Beckman DU-800, CA, USA). Meanwhile, total RNA integrity was evaluated by 1% agarose gel electrophoresis. Following this procedure, the RNA samples were used to synthesize cDNA using a PrimeScript™ RT reagent kit (TaKaRa) with the following steps: 42°C for 2 min, and then 37°C for 15 min and 85°C for 5 s.

### qPCR

Jejunal and ileal mRNA levels of NF-E2-related factor 2 (Nrf2), Kelch-like ECH-associated protein 1 (Keap1), heme oxygenase-1 (HO-1), mammalian target of rapamycin (mTOR), ribosomal p70 S6 kinase (S6K1) and eukaryotic initiation factor 4E-binding protein 1 (4EBP1) were quantified using qPCR (Cao et al., 2017). In brief, the specific primers were designed using Primer Express 3.0 software (Applied Biosystems, Foster City, CA, USA) and purchased from TsingKe Biological Technology Co., Ltd. (Chengdu, China) as depicted in Table 1. Amplification was performed in a final volume of 10 μL, which consisted of 5 μL of SYBR® *Premix Ex* Taq^TM^ II (Tli RNaseH Plus) and 0.5 μL forward and reverse primers, 1 μL cDNA and 3 μL DEPC-treated water. All qPCR reactions were performed on a CFX96 Real-Time System (Bio-Rad, Hercules, CA, USA) under the following cycling conditions: 30 s at 95°C, followed by 40 cycles at 95°C for 5 s and 60°C for 34 s. Then, the relative expression levels of target genes to β-actin (house-keeping gene) were calculated according to the 2^−ΔΔCt^ method (Livak and Schmittgen, 2001).

**Table 1:** Primer sequences for quantitative real-time PCR.

**Gene**	**Primers sequence (5^**′**^-3^**′**^)**	**Size (bp)**	**Accession no**.
Keap1^a^	• Forward: GGCTGGGATGCCTTGTAAAG • Reverse: GGGCCCATGGATTTCAGTT	57	NM_057152.2
Nrf2^b^	• Forward: CCCATTGAGGGCTGTGATCT • Reverse: GCCTTCAGTGTGCTTCTGGTT	60	NM_031789.2
HO-1^c^	• Forward: TTAAGCTGGTGATGGCCTCC • Reverse: GTGGGGCATAGACTGGGTTC	90	NM_012580.2
mTOR^d^	• Forward: CCCTGTCTTCACTTGTGTTTCAAC • Reverse: TCGTAGCGCTGGTGATTGATC	103	NM_019906.1
S6K1^e^	• Forward: AAGAGGGCTTCTCCTTCCAG • Reverse: AACCCCTCAAAGGGAGAGAA	103	xNM_001010962
4EBP1^f^	• Forward: CTAGCCCTACCAGCGATGAG • Reverse: TGGCTGGTCCCTTAAATGTC	124	NM_053857
β-Actin	• Forward: GCAAGCAGGAGTACGATGAGT • Reverse: GGTGTAAAACGCAGCTCAGTA	86	NM_007393.5

a *Keap1, Kelch-like ECH-associated protein 1*.

b *Nrf2, NF-E2-related factor 2*.

c *HO-1, heme oxygenase-1*.

d *mTOR, mammalian target of rapamycin*.

e *S6K1, ribosomal protein S6 kinase 1*.

f *4EBP1, eukaryotic initiation factor 4E-binding protein 1*.

### Statistical Analysis

All data were analyzed by Student's *t*-test using SAS 8.2 (SAS Institute, Inc., Cary, NC, USA) with each rat serving as a statistical unit. Data are shown as the mean ± standard error. *P* < 0.05 was considered statistically significant when used to compare differences between the two groups.

## Results

### Growth Performance

**Figure 1:**
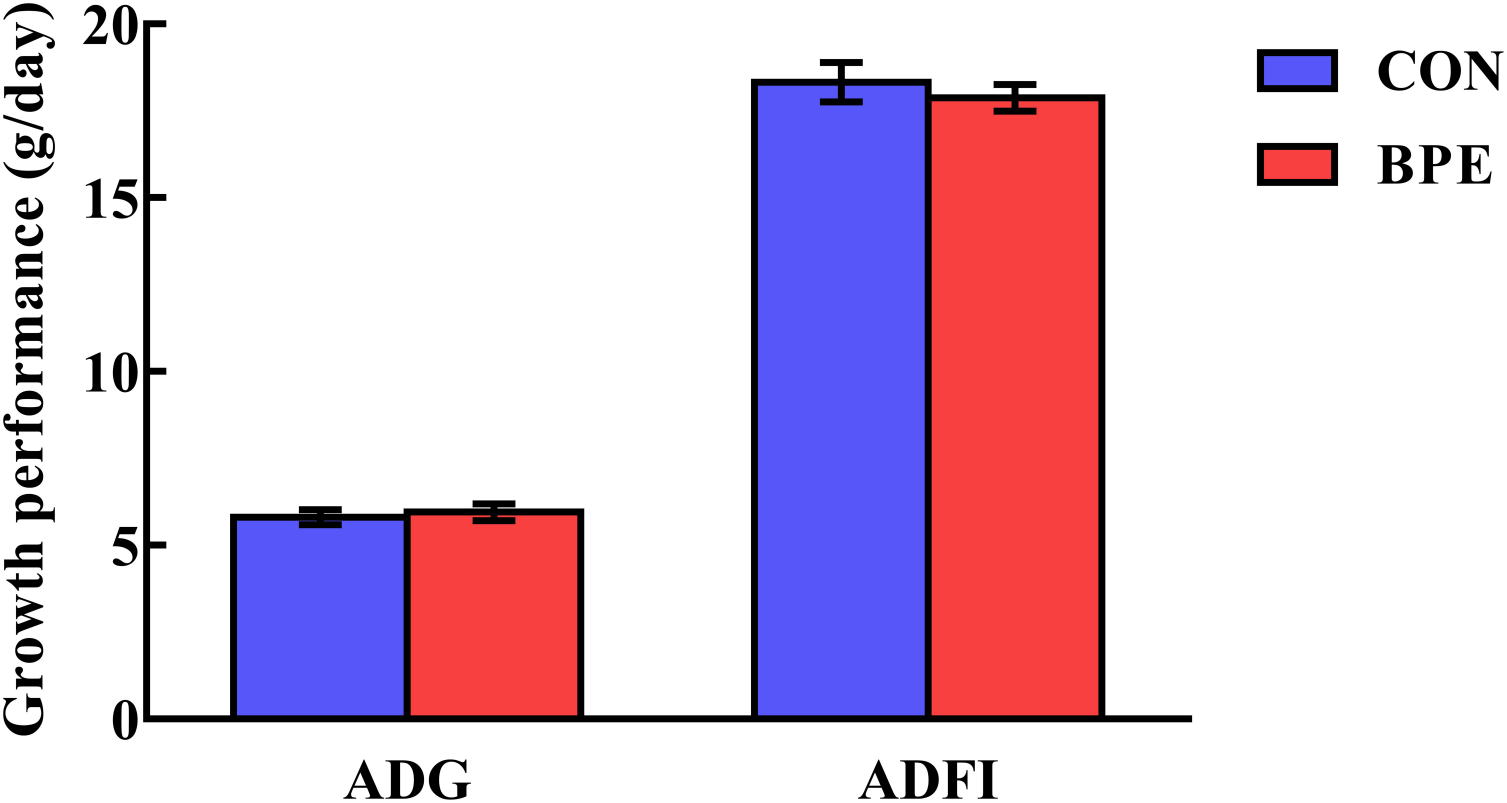
Effects of blueberry polyphenol extracts on the growth performance of weaned rats. Values are means with standard errors represented by vertical bars. ADG, average daily gain. ADFI, average daily feed intake.

The effects of BPE on the growth performance of weaned rats are shown in Figure 1. Rat growth performance was not (*P* > 0.05) impacted by the BPE supplementation during any part of the experiment.

### Antioxidant Parameters

**Figure 2:**
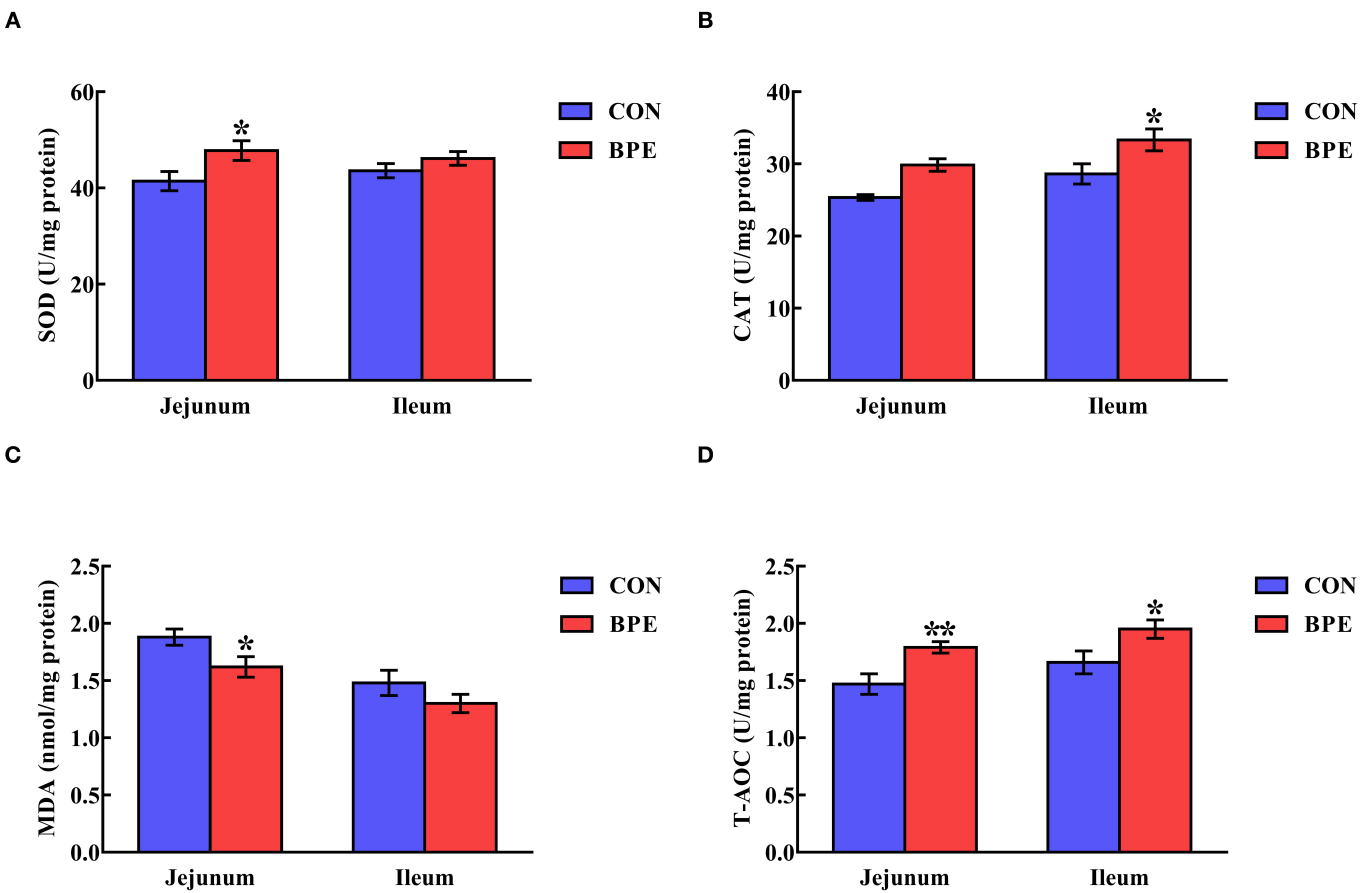
Effects of blueberry polyphenol extracts on the jejunal and ileal antioxidant status of weaned rats. **(A)** SOD, superoxide dismutase. **(B)** CAT, catalase. **(C)** MDA, malondialdehyde. **(D)** T-AOC, total antioxidant capacity. Values are means with standard errors represented by vertical bars. *P < 0.05 or **P < 0.01.

As depicted in Figure 2, dietary supplementation with BPE significantly increased (*P* < 0.05) jejunal SOD activity and ileal CAT activity by 16.17% and 16.46%, respectively. Meanwhile, the jejunal and ileal T-AOC activities were elevated (*P* < 0.05) by BPE ingestion. In comparison with the CON group, BPE supplementation decreased (*P* < 0.05) the jejunal MDA content by 13.83%.

### Cytokine Contents

**Figure 3:**
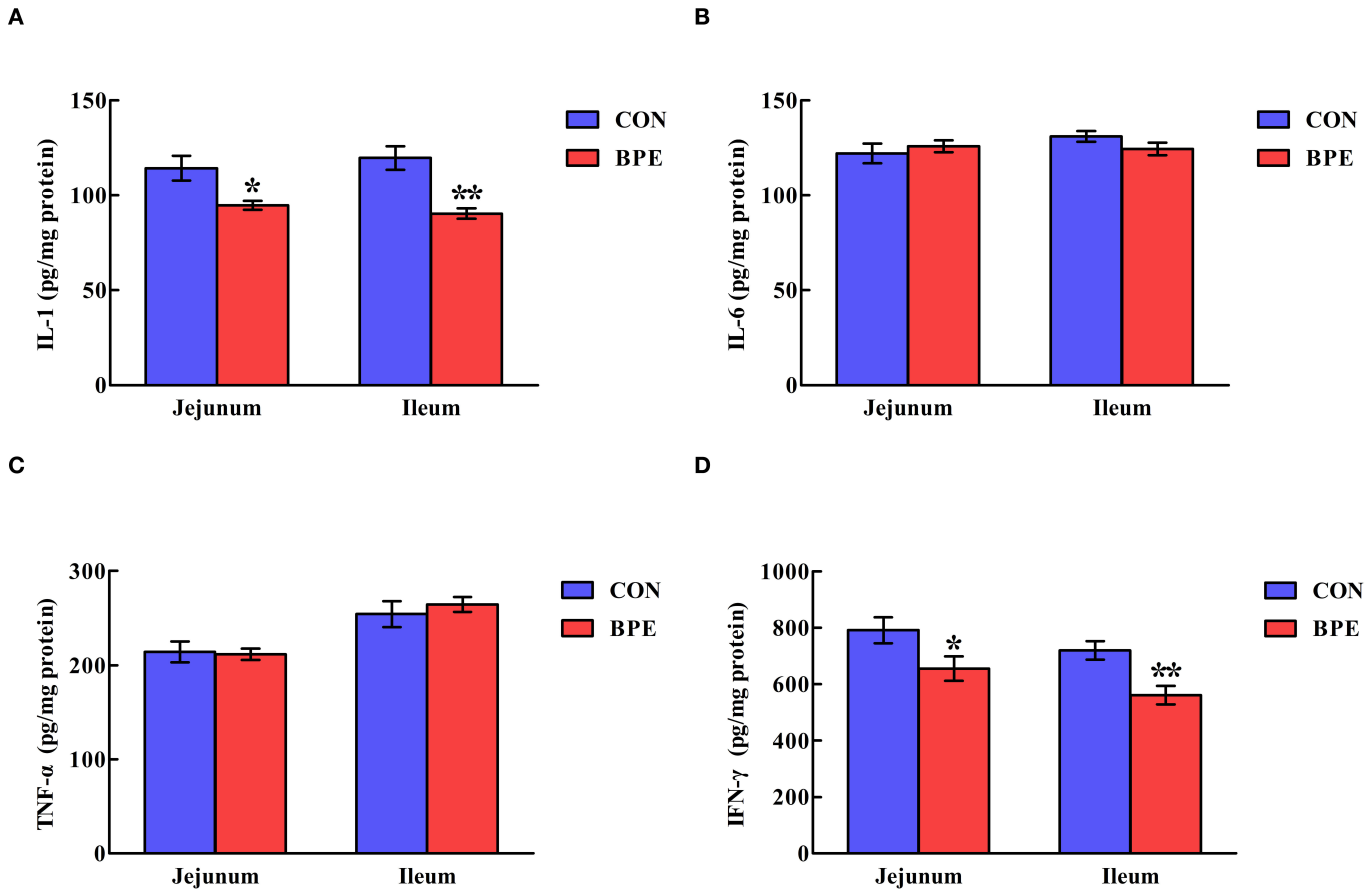
Effects of blueberry polyphenol extracts on the jejunal and ileal cytokine contents of weaned rats. **(A)** IL-1, interleukin-1. **(B)** IL-6, interleukin-6. **(C)** TNF-α, tumor necrosis factor α. **(D)** IFN-γ, interferon-γ. Values are means with standard errors represented by vertical bars. *P < 0.05 or **P < 0.01.

Figure 3 reveals that the IL-1 and IFN-γ contents in the CON group were higher (*P* < 0.05) than those in the BPE group. Nevertheless, the IL-6 and TNF-α contents were not heavily influenced (*P* > 0.05) by BPE supplementation.

### Transcription of Nrf2-Related Genes

**Figure 4:**
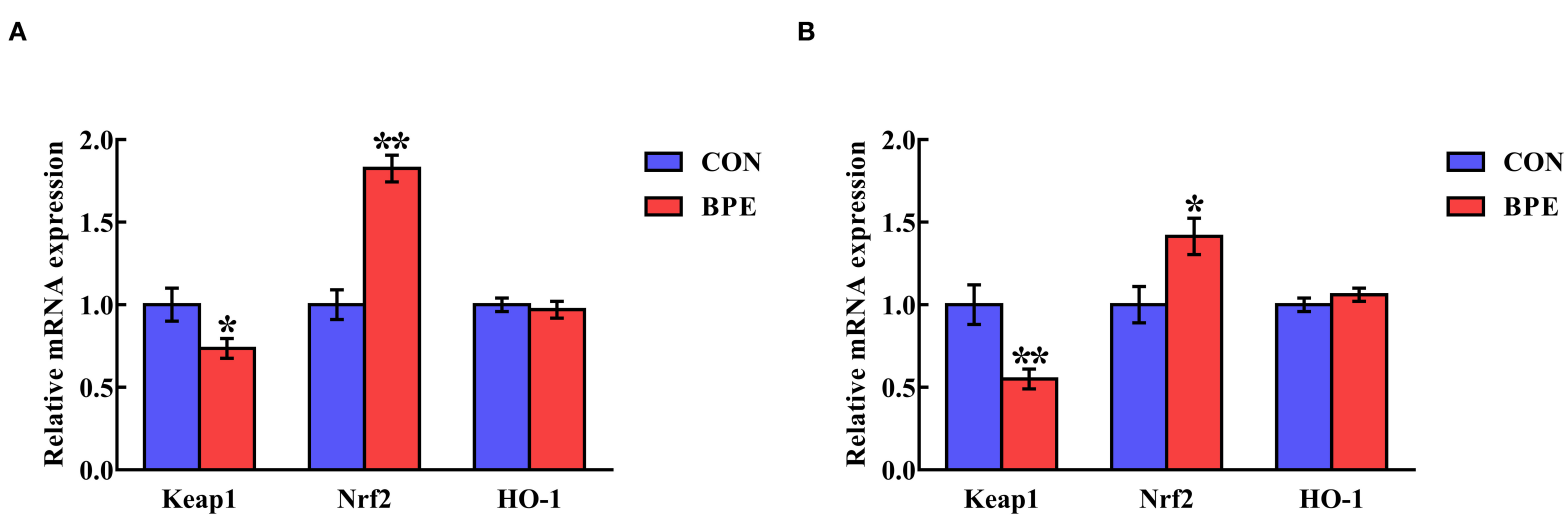
Effects of blueberry polyphenol extracts on the relative mRNA levels of Keap1 and Nrf2 in the jejunum **(A)** and ileum **(B)** of weaned rats. Values are means with standard errors represented by vertical bars. *P < 0.05 or **P < 0.01. Keap1, Kelch-like ECH-associated protein 1. Nrf2, NF-E2-related factor 2.

The influences of BPE on the jejunal and ileal mRNA levels of Keap1 and Nrf2 in weaned rats are shown in Figure 4. BPE supplementation markedly down-regulated (*P* < 0.05) Keap1 gene transcription and up-regulated (*P* < 0.05) Nrf2 gene transcription in the jejunum and ileum relative to the CON group. At the same time, there were no differences (*P* > 0.05) in the jejunal and ileal HO-1 mRNA abundances between the two groups.

### Transcription of mTOR-Related Genes

**Figure 5:**
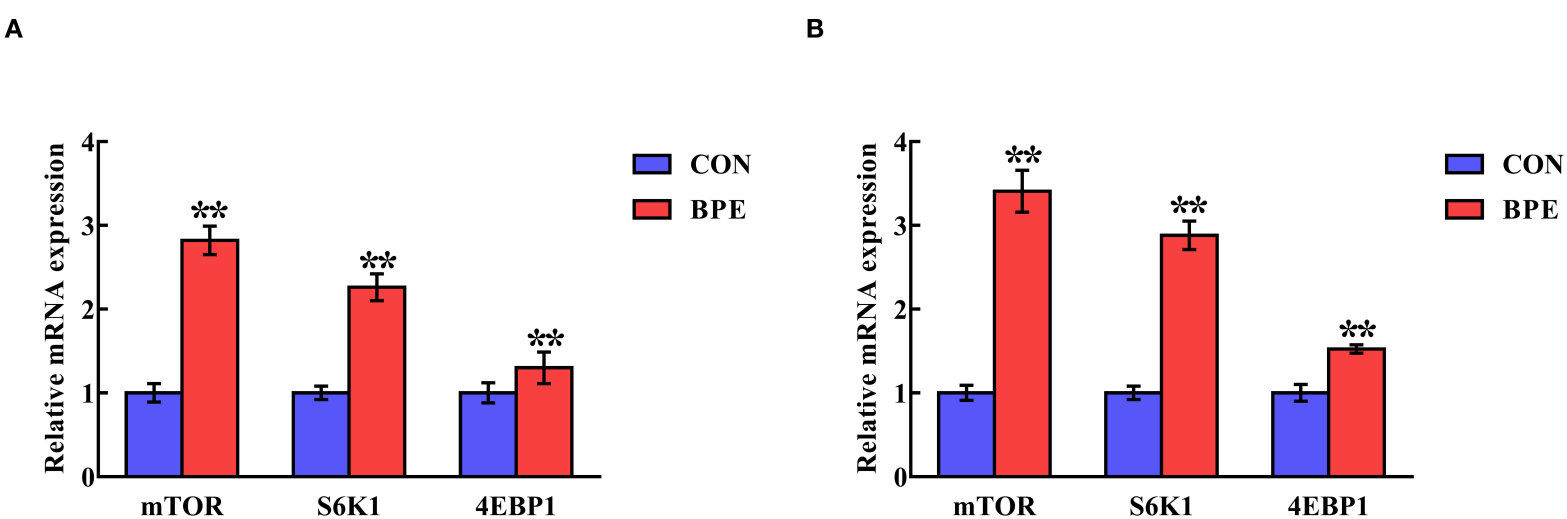
Effects of blueberry polyphenol extracts on the relative mRNA levels of mTOR, S6K1 and 4EBP1 in the jejunum **(A)** and ileum **(B)** of weaned rats. Values are means with standard errors represented by vertical bars. **P < 0.01. PI3K, phosphatidylinositol 3-kinase. mTOR, mammalian target of rapamycin. S6K1, ribosomal protein S6 kinase 1. 4EBP1, eukaryotic initiation factor 4E-binding protein 1.

Figure 5 indicates the alterations in the mRNA abundance of mTOR-related genes in the jejunum and ileum of weaned rats. BPE administration significantly up-regulated (*P* < 0.05) the mRNA abundance of mTOR, S6K1 and 4EBP1 in the jejunum and ileum compared with the control groups.

## Discussion

Organisms possess the capacity to protect themselves from oxidative stress via antioxidant defense systems involving non-enzymatic and enzymatic antioxidants (Cao et al., 2018). Weaning is associated with increased susceptibility to disease as well as impaired structure and function of the small intestine because of its poor antioxidant system (Campbell et al., 2013). The small intestine, a vital organ for mammalian health, is vulnerable to serious damage from oxidative stress. As such, maintaining the proper functioning of the antioxidant defense system in this organ is essential for animal growth and health. Previous research has demonstrated that BPE could serve as an antioxidant to mitigate oxidative stress in rats (Miyazaki et al., 2020). Therefore, we examined whether BPE supplementation could enhance the antioxidant status in the small intestine of weaned rats. Our results revealed that the jejunal MDA content was reduced by BPE ingestion, indicating that BPE can suppress lipid peroxidation in the ileum (Pirinccioglu et al., 2010). In the present work, remarkable enhancements of the activity of jejunal SOD and ileal CAT, two important endogenous antioxidant enzymes in the antioxidant defense systems, were observed. Furthermore, an enhanced T-AOC was also observed in both the jejunum and ileum after BPE supplementation, which further reflects the elevated antioxidant capacity in these organs (Cao et al., 2016). The present work also revealed that pro-inflammatory factors, such as IL-1 and IFN-γ, were decreased by BPE supplementation. Collectively, these findings suggest that BPE supplementation can protect the small intestine from oxidative stress and associated inflammation.

Given the above findings, exploring the potential mechanisms underlying the changes in antioxidant status could offer new insights into the influence of BPE ingestion on the effects of weaning. Therefore, we then evaluated the mechanisms by which BPE regulates antioxidant status in the jejunum and ileum of weaned rats. Nrf2 is regarded as an important nuclear transcription factor in accelerating endogenous antioxidant enzyme gene transcription, which is applied through binding to the antioxidant response element in the promoter region of these antioxidant enzyme genes (Kensler et al., 2007; Zhang et al., 2013). In the current study, we found that BPE supplementation up-regulated the Nrf2 transcription level in the jejunum and ileum. These results indicate that the beneficial effects of BPE supplementation on the activities of antioxidant enzymes in weaned rats are partially attributable to the up-regulation of Nrf2 mRNA abundance in the jejunum and ileum. As the cytosolic repressor of Nrf2, Keap1 can retain Nrf2 in the cytoplasm and promote Nrf2 degradation via the proteasome under normal physiological conditions (Reisman et al., 2009). Here, we also found that BPE supplementation significantly down-regulated Keap1 mRNA abundance in the jejunum and ileum. Taken together, these findings suggest that BPE supplementation may increase jejunal and ileal antioxidant capacity by regulating the Nrf2–Keap1 signaling pathway.

As noted previously, the effects of BPE supplementation on the Nrf2–Keap1 signaling pathway might be associated with the mTOR signal pathway, as mTOR is an upstream signaling molecule of Nrf2 and can regulate Nrf2 expression (Shay et al., 2012; Rhee and Bae, 2015). Therefore, to further clarify the correlation between BPE and the antioxidant capacity of weaned rats, we next investigated the impacts of BPE on the mRNA expression levels of mTOR signaling molecules in the jejunum and ileum of weaned rats. Our results showed that treatment with BPE obviously increased the mTOR mRNA level in both the jejunum and ileum, clearly suggesting that the mTOR-mediated signaling pathways were stimulated by BPE. Moreover, the current study found that the mRNA levels of S6K1 and 4EBP1, two major downstream effector molecules of the mTOR signaling pathway, were up-regulated in the jejunum and ileum (Zhou et al., 2015). Hence, the up-regulated S6K1 and 4EBP1 mRNA levels in the jejunum and ileum fit with the interpretation that Nrf2 accumulation may be a result of alterations in the mTOR signaling pathway caused by BPE. Nevertheless, the relationship between BPE and the Nrf2 and mTOR signaling pathways remains unclear and needs further investigation.

## Conclusions

We have shown that supplementing the diet of weaned rats with 200 mg/kg BPE promoted the antioxidant defense system in the small intestine through enhancing non-enzymatic and enzymatic antioxidant capacities. Furthermore, the beneficial effects of BPE on small intestinal antioxidant capacity appear to be mediated through the Nrf2–Keap1 pathway.

## Data Availability Statement

The original contributions presented in the study are included in the article/supplementary material, further inquiries can be directed to the corresponding author/s.

## Ethics Statement

The animal study was reviewed and approved by All procedures in this study were in accordance with the ethical standards, which approved by the Animal Care and Use Committee of Sichuan Agricultural University (Chengdu, China).

## Author Contributions

FZ performed the experiments and wrote the manuscript. SY assisted in data analysis. MT conceived and designed the study. All authors contributed to the article and approved the submitted version.

## Conflict of Interest

FZ was employed by company Chengdu Diversity Co. Ltd. (Chengdu, China). The remaining authors declare that the research was conducted in the absence of any commercial or financial relationships that could be construed as a potential conflict of interest.
